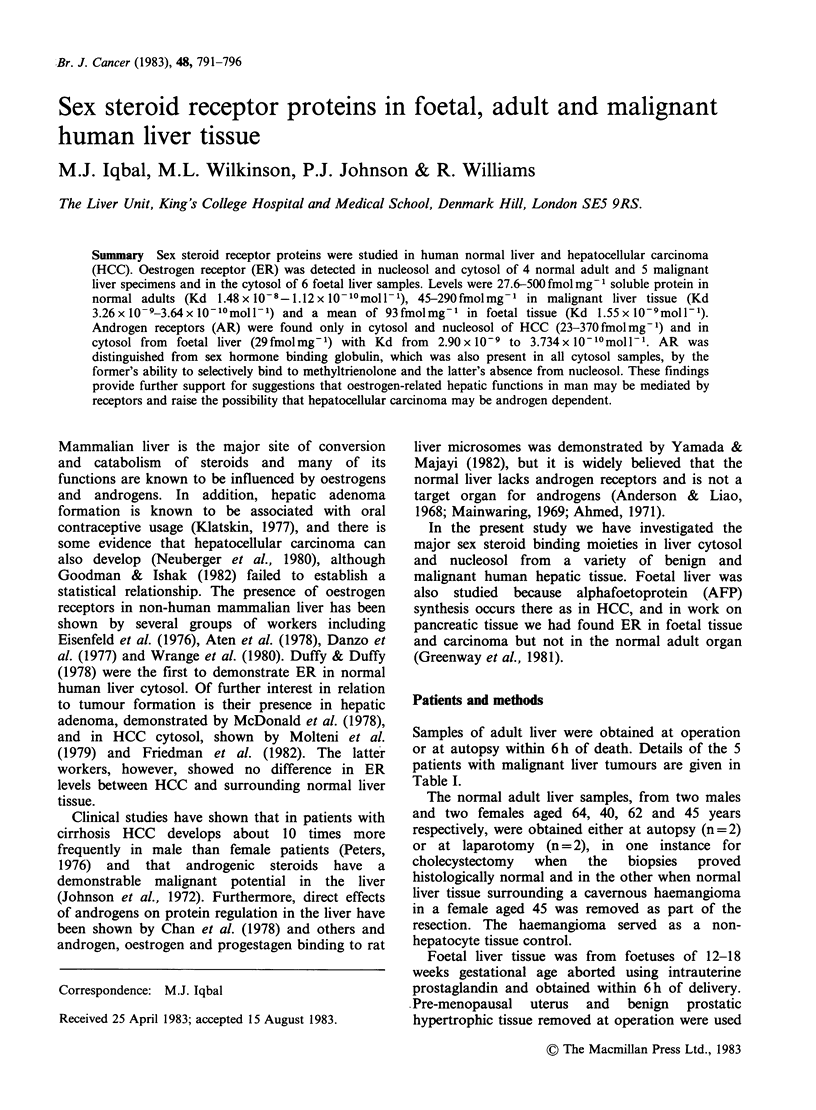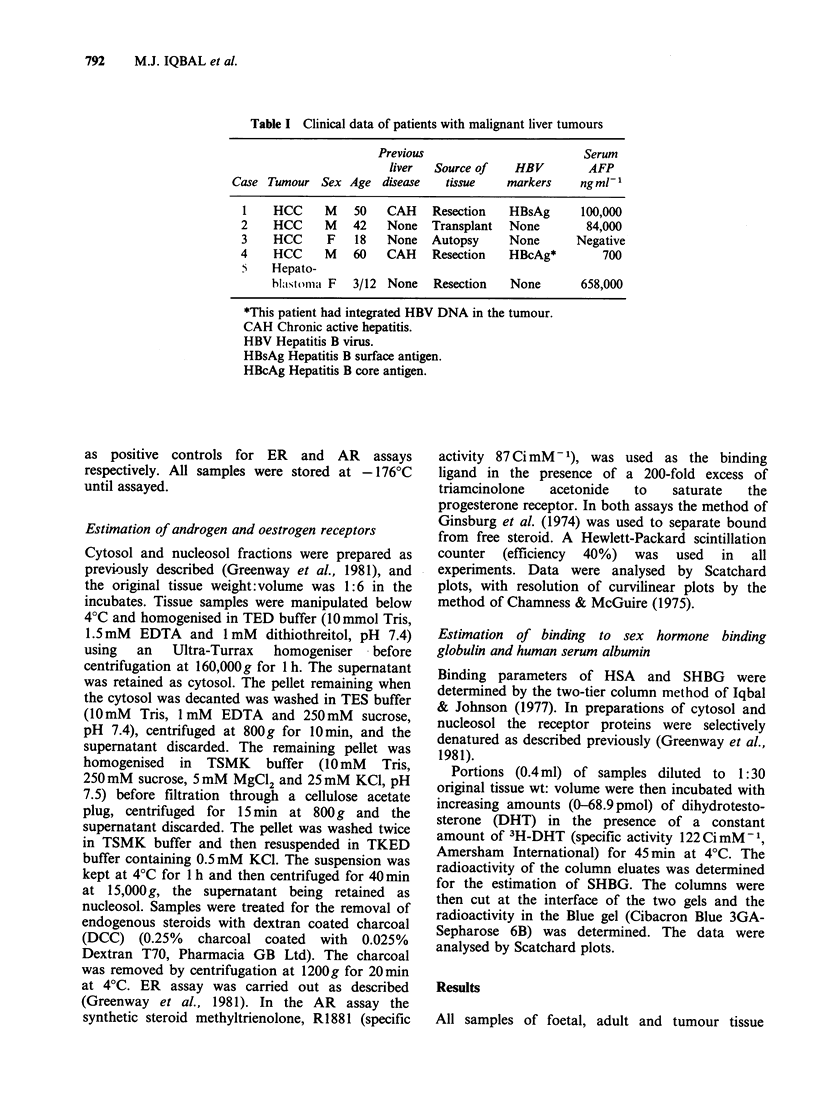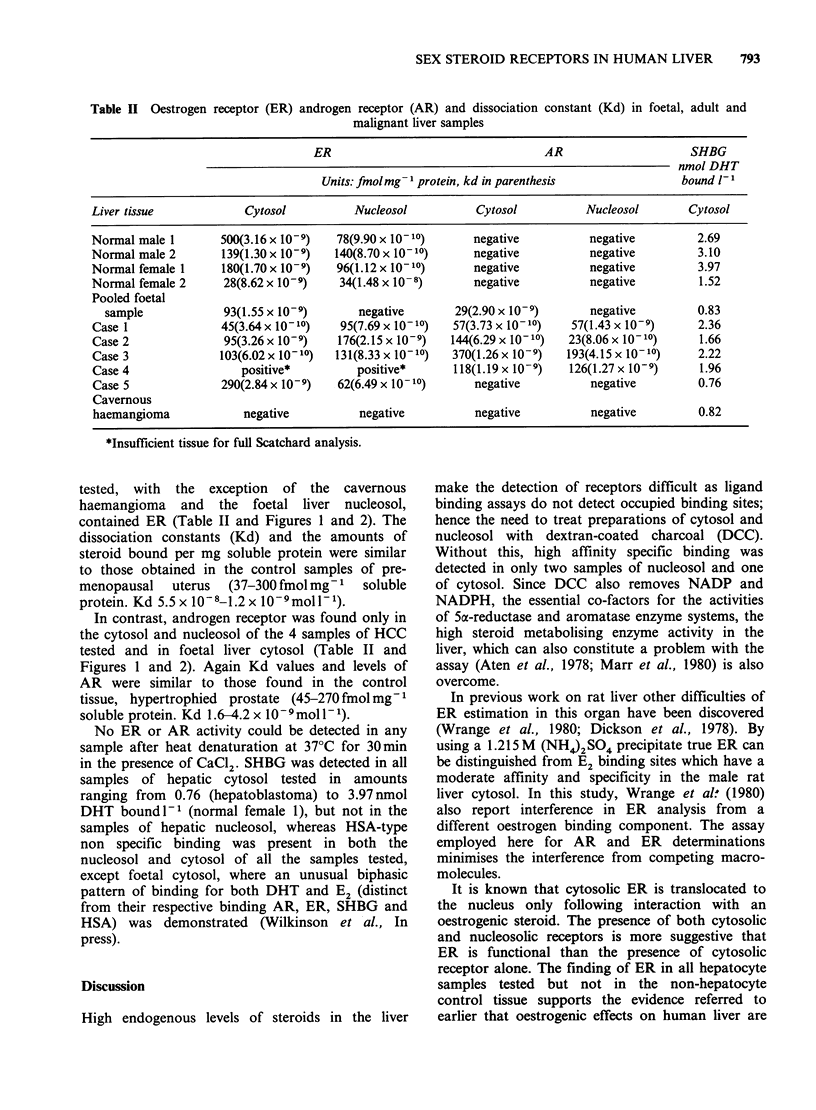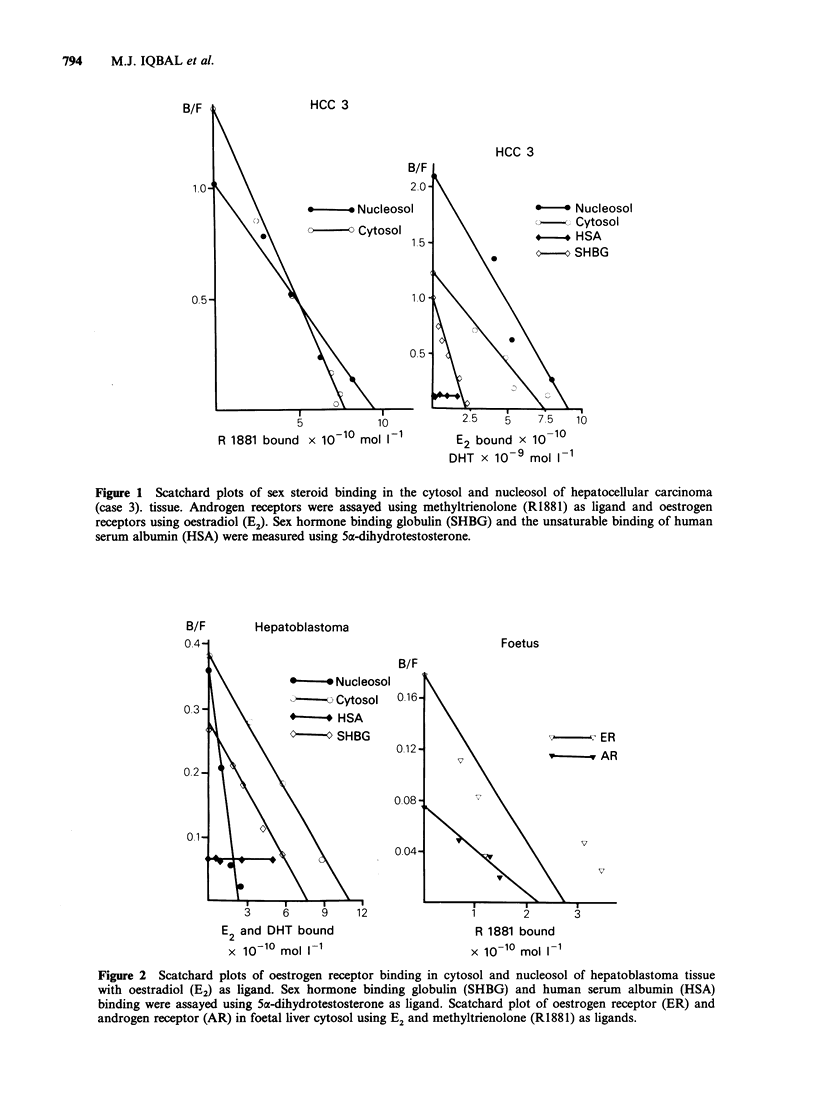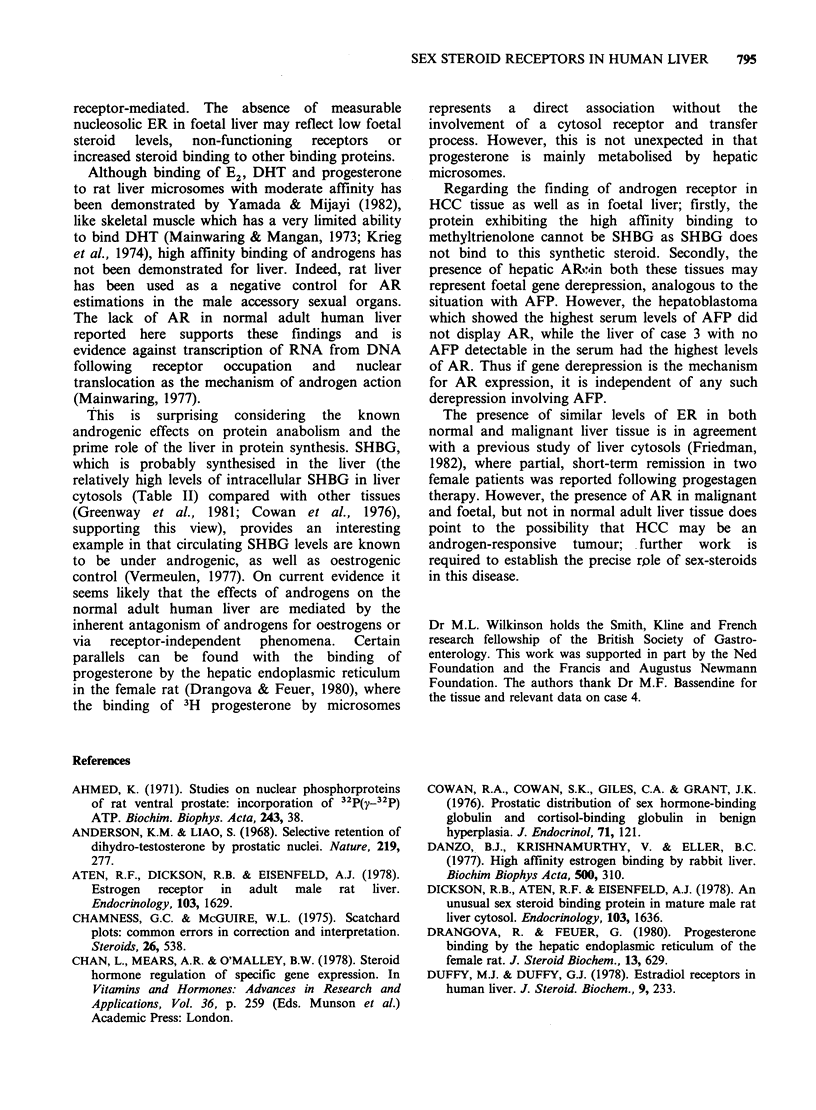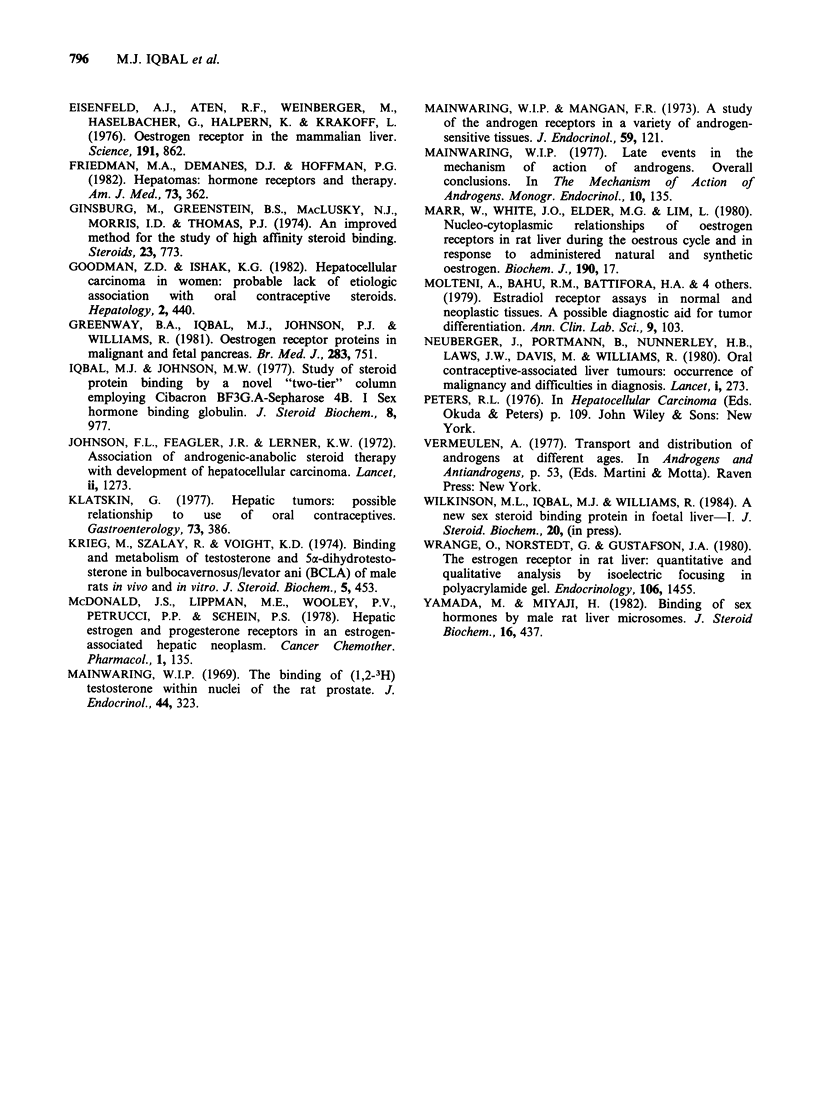# Sex steroid receptor proteins in foetal, adult and malignant human liver tissue.

**DOI:** 10.1038/bjc.1983.268

**Published:** 1983-12

**Authors:** M. J. Iqbal, M. L. Wilkinson, P. J. Johnson, R. Williams

## Abstract

Sex steroid receptor proteins were studied in human normal liver and hepatocellular carcinoma (HCC). Oestrogen receptor (ER) was detected in nucleosol and cytosol of 4 normal adult and 5 malignant liver specimens and in the cytosol of 6 foetal liver samples. Levels were 27.6-500 fmol mg-1 soluble protein in normal adults (Kd 1.48 X 10(-8) -1.12 X 10(-10) mol 1(-1) ), 45-290 fmol mg-1 in malignant liver tissue (Kd 3.26 X 10(-9) -3.64 X 10(-10) mol 1(-1] and a mean of 93 fmol mg-1 in foetal tissue (Kd 1.55 X 10(-9) mol 1(-1]. Androgen receptors (AR) were found only in cytosol and nucleosol of HCC (23-370 fmol mg-1) and in cytosol from foetal liver (29 fmol mg-1) with Kd from 2.90 X 10(-9) to 3.734 X 10(-10) mol 1(-1]. AR was distinguished from sex hormone binding globulin, which was also present in all cytosol samples, by the former's ability to selectively bind to methyltrienolone and the latter's absence from nucleosol. These findings provide further support for suggestions that oestrogen-related hepatic functions in man may be mediated by receptors and raise the possibility that hepatocellular carcinoma may be androgen dependent.


Br. J. Cancer (1983), 48, 791–796

### Sex steroid receptor proteins in foetal, adult and malignant human liver tissue

M.J. Iqbal, M.L. Wilkinson, P.J. Johnson & R. Williams

*The Liver Unit, King's College Hospital and Medical School, Denmark Hill, London SE5 9RS.*

**Summary** Sex steroid receptor proteins were studied in human normal liver and hepatocellular carcinoma (HCC). Oestrogen receptor (ER) was detected in nucleosol and cytosol of 4 normal adult and 5 malignant liver specimens and in the cytosol of 6 foetal liver samples. Levels were 27.6–500 fmol mg$^{-1}$ soluble protein in normal adults (Kd $1.48\times10^{-8}-1.12\times10^{-10}$ mol l$^{-1}$), 45–290 fmol mg$^{-1}$ in malignant liver tissue (Kd $3.26\times10^{-9}$–$3.64\times10^{-10}$ mol l$^{-1}$) and a mean of 93 fmol mg$^{-1}$ in foetal tissue (Kd $1.55\times10^{-9}$ mol l$^{-1}$). Androgen receptors (AR) were found only in cytosol and nucleosol of HCC (23–370 fmol mg$^{-1}$) and in cytosol from foetal liver (29 fmol mg$^{-1}$) with Kd from $2.90\times10^{-9}$ to $3.734\times10^{-10}$ mol l$^{-1}$. AR was distinguished from sex hormone binding globulin, which was also present in all cytosol samples, by the former's ability to selectively bind to methyltrienolone and the latter's absence from nucleosol. These findings provide further support for suggestions that oestrogen-related hepatic functions in man may be mediated by receptors and raise the possibility that hepatocellular carcinoma may be androgen dependent.

Mammalian liver is the major site of conversion and catabolism of steroids and many of its functions are known to be influenced by oestrogens and androgens. In addition, hepatic adenoma formation is known to be associated with oral contraceptive usage (Klatskin, 1977), and there is some evidence that hepatocellular carcinoma can also develop (Neuberger *et al.*, 1980), although Goodman & Ishak (1982) failed to establish a statistical relationship. The presence of oestrogen receptors in non-human mammalian liver has been shown by several groups of workers including Eisenfeld *et al.* (1976), Aten *et al.* (1978), Danzo *et al.* (1977) and Wrange *et al.* (1980). Duffy & Duffy (1978) were the first to demonstrate ER in normal human liver cytosol. Of further interest in relation to tumour formation is their presence in hepatic adenoma, demonstrated by McDonald *et al.* (1978), and in HCC cytosol, shown by Molteni *et al.* (1979) and Friedman *et al.* (1982). The latter workers, however, showed no difference in ER levels between HCC and surrounding normal liver tissue.

Clinical studies have shown that in patients with cirrhosis HCC develops about 10 times more frequently in male than female patients (Peters, 1976) and that androgenic steroids have a demonstrable malignant potential in the liver (Johnson *et al.*, 1972). Furthermore, direct effects of androgens on protein regulation in the liver have been shown by Chan *et al.* (1978) and others and androgen, oestrogen and progestagen binding to rat liver microsomes was demonstrated by Yamada & Majayi (1982), but it is widely believed that the normal liver lacks androgen receptors and is not a target organ for androgens (Anderson & Liao, 1968; Mainwaring, 1969; Ahmed, 1971).

In the present study we have investigated the major sex steroid binding moieties in liver cytosol and nucleosol from a variety of benign and malignant human hepatic tissue. Foetal liver was also studied because alphafoetoprotein (AFP) synthesis occurs there as in HCC, and in work on pancreatic tissue we had found ER in foetal tissue and carcinoma but not in the normal adult organ (Greenway *et al.*, 1981).

### Patients and methods

Samples of adult liver were obtained at operation or at autopsy within 6 h of death. Details of the 5 patients with malignant liver tumours are given in Table I.

The normal adult liver samples, from two males and two females aged 64, 40, 62 and 45 years respectively, were obtained either at autopsy (n = 2) or at laparotomy (n = 2), in one instance for cholecystectomy when the biopsies proved histologically normal and in the other when normal liver tissue surrounding a cavernous haemangioma in a female aged 45 was removed as part of the resection. The haemangioma served as a non-hepatocyte tissue control.

Foetal liver tissue was from foetuses of 12–18 weeks gestational age aborted using intrauterine prostaglandin and obtained within 6 h of delivery. Pre-menopausal uterus and benign prostatic hypertrophic tissue removed at operation were used

Correspondence: M.J. Iqbal

Received 25 April 1983; accepted 15 August 1983.

© The Macmillan Press Ltd., 1983

**Table I** Clinical data of patients with malignant liver tumours

| *Case* | *Tumour* | *Sex* | *Age* | *Previous liver disease* | *Source of tissue* | *HBV markers* | *Serum AFP ng ml$^{-1}$* |
|---|---|---|---|---|---|---|---|
| 1 | HCC | M | 50 | CAH | Resection | HBsAg | 100,000 |
| 2 | HCC | M | 42 | None | Transplant | None | 84,000 |
| 3 | HCC | F | 18 | None | Autopsy | None | Negative |
| 4 | HCC | M | 60 | CAH | Resection | HBcAg* | 700 |
| 5 | Hepato-blastoma | F | 3/12 | None | Resection | None | 658,000 |

*This patient had integrated HBV DNA in the tumour.
CAH Chronic active hepatitis.
HBV Hepatitis B virus.
HBsAg Hepatitis B surface antigen.
HBcAg Hepatitis B core antigen.

as positive controls for ER and AR assays respectively. All samples were stored at −176°C until assayed.

*Estimation of androgen and oestrogen receptors*

Cytosol and nucleosol fractions were prepared as previously described (Greenway *et al.*, 1981), and the original tissue weight:volume was 1:6 in the incubates. Tissue samples were manipulated below 4°C and homogenised in TED buffer (10 mmol Tris, 1.5 mM EDTA and 1 mM dithiothreitol, pH 7.4) using an Ultra-Turrax homogeniser before centrifugation at 160,000 *g* for 1 h. The supernatant was retained as cytosol. The pellet remaining when the cytosol was decanted was washed in TES buffer (10 mM Tris, 1 mM EDTA and 250 mM sucrose, pH 7.4), centrifuged at 800 *g* for 10 min, and the supernatant discarded. The remaining pellet was homogenised in TSMK buffer (10 mM Tris, 250 mM sucrose, 5 mM $MgCl_2$ and 25 mM KCl, pH 7.5) before filtration through a cellulose acetate plug, centrifuged for 15 min at 800 *g* and the supernatant discarded. The pellet was washed twice in TSMK buffer and then resuspended in TKED buffer containing 0.5 mM KCl. The suspension was kept at 4°C for 1 h and then centrifuged for 40 min at 15,000 *g*, the supernatant being retained as nucleosol. Samples were treated for the removal of endogenous steroids with dextran coated charcoal (DCC) (0.25% charcoal coated with 0.025% Dextran T70, Pharmacia GB Ltd). The charcoal was removed by centrifugation at 1200 *g* for 20 min at 4°C. ER assay was carried out as described (Greenway *et al.*, 1981). In the AR assay the synthetic steroid methyltrienolone, R1881 (specific activity 87 Ci mM$^{-1}$), was used as the binding ligand in the presence of a 200-fold excess of triamcinolone acetonide to saturate the progesterone receptor. In both assays the method of Ginsburg *et al.* (1974) was used to separate bound from free steroid. A Hewlett-Packard scintillation counter (efficiency 40%) was used in all experiments. Data were analysed by Scatchard plots, with resolution of curvilinear plots by the method of Chamness & McGuire (1975).

*Estimation of binding to sex hormone binding globulin and human serum albumin*

Binding parameters of HSA and SHBG were determined by the two-tier column method of Iqbal & Johnson (1977). In preparations of cytosol and nucleosol the receptor proteins were selectively denatured as described previously (Greenway *et al.*, 1981).

Portions (0.4 ml) of samples diluted to 1:30 original tissue wt: volume were then incubated with increasing amounts (0–68.9 pmol) of dihydrotestosterone (DHT) in the presence of a constant amount of $^3$H-DHT (specific activity 122 Ci mM$^{-1}$, Amersham International) for 45 min at 4°C. The radioactivity of the column eluates was determined for the estimation of SHBG. The columns were then cut at the interface of the two gels and the radioactivity in the Blue gel (Cibacron Blue 3GA-Sepharose 6B) was determined. The data were analysed by Scatchard plots.

### Results

All samples of foetal, adult and tumour tissue

**Table II** Oestrogen receptor (ER) androgen receptor (AR) and dissociation constant (Kd) in foetal, adult and malignant liver samples

| | *ER* | | *AR* | | *SHBG nmol DHT bound $l^{-1}$* |
|---|---|---|---|---|---|
| | *Units: fmol $mg^{-1}$ protein, kd in parenthesis* | | | | |
| *Liver tissue* | *Cytosol* | *Nucleosol* | *Cytosol* | *Nucleosol* | *Cytosol* |
| Normal male 1 | $500(3.16 \times 10^{-9})$ | $78(9.90 \times 10^{-10})$ | negative | negative | 2.69 |
| Normal male 2 | $139(1.30 \times 10^{-9})$ | $140(8.70 \times 10^{-10})$ | negative | negative | 3.10 |
| Normal female 1 | $180(1.70 \times 10^{-9})$ | $96(1.12 \times 10^{-10})$ | negative | negative | 3.97 |
| Normal female 2 | $28(8.62 \times 10^{-9})$ | $34(1.48 \times 10^{-8})$ | negative | negative | 1.52 |
| Pooled foetal sample | $93(1.55 \times 10^{-9})$ | negative | $29(2.90 \times 10^{-9})$ | negative | 0.83 |
| Case 1 | $45(3.64 \times 10^{-10})$ | $95(7.69 \times 10^{-10})$ | $57(3.73 \times 10^{-10})$ | $57(1.43 \times 10^{-9})$ | 2.36 |
| Case 2 | $95(3.26 \times 10^{-9})$ | $176(2.15 \times 10^{-9})$ | $144(6.29 \times 10^{-10})$ | $23(8.06 \times 10^{-10})$ | 1.66 |
| Case 3 | $103(6.02 \times 10^{-10})$ | $131(8.33 \times 10^{-10})$ | $370(1.26 \times 10^{-9})$ | $193(4.15 \times 10^{-10})$ | 2.22 |
| Case 4 | positive* | positive* | $118(1.19 \times 10^{-9})$ | $126(1.27 \times 10^{-9})$ | 1.96 |
| Case 5 | $290(2.84 \times 10^{-9})$ | $62(6.49 \times 10^{-10})$ | negative | negative | 0.76 |
| Cavernous haemangioma | negative | negative | negative | negative | 0.82 |

*Insufficient tissue for full Scatchard analysis.

tested, with the exception of the cavernous haemangioma and the foetal liver nucleosol, contained ER (Table II and Figures 1 and 2). The dissociation constants (Kd) and the amounts of steroid bound per mg soluble protein were similar to those obtained in the control samples of pre-menopausal uterus (37–300 fmol $mg^{-1}$ soluble protein. Kd $5.5 \times 10^{-8}$–$1.2 \times 10^{-9}$ mol $l^{-1}$).

In contrast, androgen receptor was found only in the cytosol and nucleosol of the 4 samples of HCC tested and in foetal liver cytosol (Table II and Figures 1 and 2). Again Kd values and levels of AR were similar to those found in the control tissue, hypertrophied prostate (45–270 fmol $mg^{-1}$ soluble protein. Kd 1.6–$4.2 \times 10^{-9}$ mol $l^{-1}$).

No ER or AR activity could be detected in any sample after heat denaturation at 37°C for 30 min in the presence of $CaCl_2$. SHBG was detected in all samples of hepatic cytosol tested in amounts ranging from 0.76 (hepatoblastoma) to 3.97 nmol DHT bound $l^{-1}$ (normal female 1), but not in the samples of hepatic nucleosol, whereas HSA-type non specific binding was present in both the nucleosol and cytosol of all the samples tested, except foetal cytosol, where an unusual biphasic pattern of binding for both DHT and $E_2$ (distinct from their respective binding AR, ER, SHBG and HSA) was demonstrated (Wilkinson *et al.*, In press).

### Discussion

High endogenous levels of steroids in the liver make the detection of receptors difficult as ligand binding assays do not detect occupied binding sites; hence the need to treat preparations of cytosol and nucleosol with dextran-coated charcoal (DCC). Without this, high affinity specific binding was detected in only two samples of nucleosol and one of cytosol. Since DCC also removes NADP and NADPH, the essential co-factors for the activities of 5α-reductase and aromatase enzyme systems, the high steroid metabolising enzyme activity in the liver, which can also constitute a problem with the assay (Aten *et al.*, 1978; Marr *et al.*, 1980) is also overcome.

In previous work on rat liver other difficulties of ER estimation in this organ have been discovered (Wrange *et al.*, 1980; Dickson *et al.*, 1978). By using a 1.215 M $(NH_4)_2SO_4$ precipitate true ER can be distinguished from $E_2$ binding sites which have a moderate affinity and specificity in the male rat liver cytosol. In this study, Wrange *et al*: (1980) also report interference in ER analysis from a different oestrogen binding component. The assay employed here for AR and ER determinations minimises the interference from competing macromolecules.

It is known that cytosolic ER is translocated to the nucleus only following interaction with an oestrogenic steroid. The presence of both cytosolic and nucleosolic receptors is more suggestive that ER is functional than the presence of cytosolic receptor alone. The finding of ER in all hepatocyte samples tested but not in the non-hepatocyte control tissue supports the evidence referred to earlier that oestrogenic effects on human liver are

**Figure 1** Scatchard plots of sex steroid binding in the cytosol and nucleosol of hepatocellular carcinoma (case 3). tissue. Androgen receptors were assayed using methyltrienolone (R1881) as ligand and oestrogen receptors using oestradiol ($E_2$). Sex hormone binding globulin (SHBG) and the unsaturable binding of human serum albumin (HSA) were measured using 5α-dihydrotestosterone.

**Figure 2** Scatchard plots of oestrogen receptor binding in cytosol and nucleosol of hepatoblastoma tissue with oestradiol ($E_2$) as ligand. Sex hormone binding globulin (SHBG) and human serum albumin (HSA) binding were assayed using 5α-dihydrotestosterone as ligand. Scatchard plot of oestrogen receptor (ER) and androgen receptor (AR) in foetal liver cytosol using $E_2$ and methyltrienolone (R1881) as ligands.

receptor-mediated. The absence of measurable nucleosolic ER in foetal liver may reflect low foetal steroid levels, non-functioning receptors or increased steroid binding to other binding proteins.

Although binding of $E_2$, DHT and progesterone to rat liver microsomes with moderate affinity has been demonstrated by Yamada & Mijayi (1982), like skeletal muscle which has a very limited ability to bind DHT (Mainwaring & Mangan, 1973; Krieg *et al.*, 1974), high affinity binding of androgens has not been demonstrated for liver. Indeed, rat liver has been used as a negative control for AR estimations in the male accessory sexual organs. The lack of AR in normal adult human liver reported here supports these findings and is evidence against transcription of RNA from DNA following receptor occupation and nuclear translocation as the mechanism of androgen action (Mainwaring, 1977).

This is surprising considering the known androgenic effects on protein anabolism and the prime role of the liver in protein synthesis. SHBG, which is probably synthesised in the liver (the relatively high levels of intracellular SHBG in liver cytosols (Table II) compared with other tissues (Greenway *et al.*, 1981; Cowan *et al.*, 1976), supporting this view), provides an interesting example in that circulating SHBG levels are known to be under androgenic, as well as oestrogenic control (Vermeulen, 1977). On current evidence it seems likely that the effects of androgens on the normal adult human liver are mediated by the inherent antagonism of androgens for oestrogens or via receptor-independent phenomena. Certain parallels can be found with the binding of progesterone by the hepatic endoplasmic reticulum in the female rat (Drangova & Feuer, 1980), where the binding of $^{3}$H progesterone by microsomes represents a direct association without the involvement of a cytosol receptor and transfer process. However, this is not unexpected in that progesterone is mainly metabolised by hepatic microsomes.

Regarding the finding of androgen receptor in HCC tissue as well as in foetal liver; firstly, the protein exhibiting the high affinity binding to methyltrienolone cannot be SHBG as SHBG does not bind to this synthetic steroid. Secondly, the presence of hepatic AR in both these tissues may represent foetal gene derepression, analogous to the situation with AFP. However, the hepatoblastoma which showed the highest serum levels of AFP did not display AR, while the liver of case 3 with no AFP detectable in the serum had the highest levels of AR. Thus if gene derepression is the mechanism for AR expression, it is independent of any such derepression involving AFP.

The presence of similar levels of ER in both normal and malignant liver tissue is in agreement with a previous study of liver cytosols (Friedman, 1982), where partial, short-term remission in two female patients was reported following progestagen therapy. However, the presence of AR in malignant and foetal, but not in normal adult liver tissue does point to the possibility that HCC may be an androgen-responsive tumour; further work is required to establish the precise role of sex-steroids in this disease.

Dr M.L. Wilkinson holds the Smith, Kline and French research fellowship of the British Society of Gastroenterology. This work was supported in part by the Ned Foundation and the Francis and Augustus Newmann Foundation. The authors thank Dr M.F. Bassendine for the tissue and relevant data on case 4.

### References

AHMED, K. (1971). Studies on nuclear phosphorproteins of rat ventral prostate: incorporation of $^{32}P(\gamma-^{32}P)$ ATP. *Biochim. Biophys. Acta*, **243**, 38.

ANDERSON, K.M. & LIAO, S. (1968). Selective retention of dihydro-testosterone by prostatic nuclei. *Nature*, **219**, 277.

ATEN, R.F., DICKSON, R.B. & EISENFELD, A.J. (1978). Estrogen receptor in adult male rat liver. *Endocrinology*, **103**, 1629.

CHAMNESS, G.C. & MCGUIRE, W.L. (1975). Scatchard plots: common errors in correction and interpretation. *Steroids*, **26**, 538.

CHAN, L., MEARS, A.R. & O'MALLEY, B.W. (1978). Steroid hormone regulation of specific gene expression. In *Vitamins and Hormones: Advances in Research and Applications, Vol. 36*, p. 259 (Eds. Munson *et al.*) Academic Press: London.

COWAN, R.A., COWAN, S.K., GILES, C.A. & GRANT, J.K. (1976). Prostatic distribution of sex hormone-binding globulin and cortisol-binding globulin in benign hyperplasia. *J. Endocrinol*, **71**, 121.

DANZO, B.J., KRISHNAMURTHY, V. & ELLER, B.C. (1977). High affinity estrogen binding by rabbit liver. *Biochim Biophys Acta*, **500**, 310.

DICKSON, R.B., ATEN, R.F. & EISENFELD, A.J. (1978). An unusual sex steroid binding protein in mature male rat liver cytosol. *Endocrinology*, **103**, 1636.

DRANGOVA, R. & FEUER, G. (1980). Progesterone binding by the hepatic endoplasmic reticulum of the female rat. *J. Steroid Biochem.*, **13**, 629.

DUFFY, M.J. & DUFFY, G.J. (1978). Estradiol receptors in human liver. *J. Steroid. Biochem.*, **9**, 233.

EISENFELD, A.J., ATEN, R.F., WEINBERGER, M., HASELBACHER, G., HALPERN, K. & KRAKOFF, L. (1976). Oestrogen receptor in the mammalian liver. *Science*, **191**, 862.

FRIEDMAN, M.A., DEMANES, D.J. & HOFFMAN, P.G. (1982). Hepatomas: hormone receptors and therapy. *Am. J. Med.*, **73**, 362.

GINSBURG, M., GREENSTEIN, B.S., MACLUSKY, N.J., MORRIS, I.D. & THOMAS, P.J. (1974). An improved method for the study of high affinity steroid binding. *Steroids*, **23**, 773.

GOODMAN, Z.D. & ISHAK, K.G. (1982). Hepatocellular carcinoma in women: probable lack of etiologic association with oral contraceptive steroids. *Hepatology*, **2**, 440.

GREENWAY, B.A., IQBAL, M.J., JOHNSON, P.J. & WILLIAMS, R. (1981). Oestrogen receptor proteins in malignant and fetal pancreas. *Br. Med. J.*, **283**, 751.

IQBAL, M.J. & JOHNSON, M.W. (1977). Study of steroid protein binding by a novel "two-tier" column employing Cibacron BF3G.A-Sepharose 4B. I Sex hormone binding globulin. *J. Steroid Biochem.*, **8**, 977.

JOHNSON, F.L., FEAGLER, J.R. & LERNER, K.W. (1972). Association of androgenic-anabolic steroid therapy with development of hepatocellular carcinoma. *Lancet*, **ii**, 1273.

KLATSKIN, G. (1977). Hepatic tumors: possible relationship to use of oral contraceptives. *Gastroenterology*, **73**, 386.

KRIEG, M., SZALAY, R. & VOIGHT, K.D. (1974). Binding and metabolism of testosterone and 5α-dihydrotestosterone in bulbocavernosus/levator ani (BCLA) of male rats *in vivo* and *in vitro*. *J. Steroid. Biochem.*, **5**, 453.

McDONALD, J.S., LIPPMAN, M.E., WOOLEY, P.V., PETRUCCI, P.P. & SCHEIN, P.S. (1978). Hepatic estrogen and progesterone receptors in an estrogen-associated hepatic neoplasm. *Cancer Chemother. Pharmacol.*, **1**, 135.

MAINWARING, W.I.P. (1969). The binding of (1,2-$^3$H) testosterone within nuclei of the rat prostate. *J. Endocrinol.*, **44**, 323.

MAINWARING, W.I.P. & MANGAN, F.R. (1973). A study of the androgen receptors in a variety of androgen-sensitive tissues. *J. Endocrinol.*, **59**, 121.

MAINWARING, W.I.P. (1977). Late events in the mechanism of action of androgens. Overall conclusions. In *The Mechanism of Action of Androgens. Monogr. Endocrinol.*, **10**, 135.

MARR, W., WHITE, J.O., ELDER, M.G. & LIM, L. (1980). Nucleo-cytoplasmic relationships of oestrogen receptors in rat liver during the oestrous cycle and in response to administered natural and synthetic oestrogen. *Biochem. J.*, **190**, 17.

MOLTENI, A., BAHU, R.M., BATTIFORA, H.A. & 4 others. (1979). Estradiol receptor assays in normal and neoplastic tissues. A possible diagnostic aid for tumor differentiation. *Ann. Clin. Lab. Sci.*, **9**, 103.

NEUBERGER, J., PORTMANN, B., NUNNERLEY, H.B., LAWS, J.W., DAVIS, M. & WILLIAMS, R. (1980). Oral contraceptive-associated liver tumours: occurrence of malignancy and difficulties in diagnosis. *Lancet*, **i**, 273.

PETERS, R.L. (1976). In *Hepatocellular Carcinoma* (Eds. Okuda & Peters) p. 109. John Wiley & Sons: New York.

VERMEULEN, A. (1977). Transport and distribution of androgens at different ages. In *Androgens and Antiandrogens*, p. 53, (Eds. Martini & Motta). Raven Press: New York.

WILKINSON, M.L., IQBAL, M.J. & WILLIAMS, R. (1984). A new sex steroid binding protein in foetal liver—I. *J. Steroid. Biochem.*, **20**, (in press).

WRANGE, O., NORSTEDT, G. & GUSTAFSON, J.A. (1980). The estrogen receptor in rat liver: quantitative and qualitative analysis by isoelectric focusing in polyacrylamide gel. *Endocrinology*, **106**, 1455.

YAMADA, M. & MIYAJI, H. (1982). Binding of sex hormones by male rat liver microsomes. *J. Steroid Biochem.*, **16**, 437.